# Novel multifibrillar carbon and oxidation-stable carbon/ceramic hybrid fibers consisting of thousands of individual nanofibers with high tensile strength

**DOI:** 10.1038/s41598-024-68794-w

**Published:** 2024-08-05

**Authors:** Jakob Denk, Xiaojian Liao, Wolfgang Knolle, Axel Kahnt, Andreas Greiner, Stefan Schafföner, Seema Agarwal, Günter Motz

**Affiliations:** 1 Chair of Ceramic Materials Engineering, University of Bayreuth, 95440 Bayreuth, Germany; 2 Macromolecular Chemistry 2 and Bavarian Polymer Institute, University of Bayreuth, 95440 Bayreuth, Germany; 3 Bavarian Center for Battery Technology (BayBatt), University of Bayreuth, 95440 Bayreuth, Germany; 4 Leibniz Institute of Surface Engineering (IOM), Permoserstr. 15, 04318 Leipzig, Germany

**Keywords:** Carbon fiber, Ceramic fiber, Electrospun multifibrillar fiber, Oligosilazane, High strength, Electron beam treatment, Chemistry, Engineering, Materials science, Nanoscience and technology

## Abstract

In this study, multifibrillar carbon and carbon/ceramic (C/SiCON) fibers consisting of thousands of single nanofibers are continuously manufactured. The process starts with electrospinning of polyacrylonitrile (PAN) and PAN/oligosilazane precursors resulting in poorly aligned polymer fibers. Subsequent stretching leads to parallel aligned multifibrillar fibers, which are continuously stabilized and pyrolyzed to C or C/SiCON hybrid fibers. The multifibrillar carbon fibers show a high tensile strength of 911 MPa and Young’s modulus of 154 GPa, whereas the multifibrillar C/SiCON fibers initially have only tensile strengths of 407 MPa and Young’s modulus of 77 GPa, due to sticking of the nanofibers during the stabilization in air. Additional curing with electron beam radiation, results in a remarkable increase in tensile strength of 707 MPa and Young's modulus of 98 GPa. The good mechanical properties are highlighted by the low linear density of the multifibrillar C/SiCON fibers (~ 1 tex) compared to conventional C and SiC fiber bundles (~ 200 tex). In combination with the large surface area of the fibers better mechanical properties of respective composites with a reduced fiber content can be achieved. In addition, the developed approach offers high potential to produce advanced endless multifibrillar carbon and C/SiCON nanofibers in an industrial scale.

## Introduction

Carbon fibers are one of the most important technical fibers. They are indispensable in numerous technical applications, for example in aviation, automotive, military technology, aerospace, medical, and sport products^1,2^. The commercial processing of these fibers is mostly performed by wet-spinning of polyacrylonitrile (PAN). Usually, PAN is dissolved in *N,N’*-dimethylformamide (DMF) or dimethyl sulfoxide (DMSO) and extruded into a precipitation bath, in which it is insoluble. The fibers obtained are then drawn, dried, and wound up^1,3^. Afterwards, thermal stabilization is carried out under tensile stress at 200–300 °C in air. During this process, the linear macromolecules form a cyclic ladder-like structure^4,5^. Then these stabilized fibers are carbonized at above 1000 °C in an inert atmosphere and are converted into carbon fibers. For high tensile carbon fibers, the pyrolysis occurs up to 1600 °C. High-modulus fibers are graphitized up to 3000 °C, whereby the tensile strength decreases^6,7^.

Carbon fibers are interesting for many applications due to their extraordinary mechanical properties^1,8^. For example, TORAY™ T1100 carbon fibers, achieve a tensile strength of 7 GPa at a low density of 1.79 g cm^−3^^9^. Despite these exceptional properties, commercial carbon fibers have only a fraction of the theoretical value. According to theoretical calculations, the tensile strength could be as high as 180 GPa^10,11^. However, for multiwalled carbon nanotubes (CNTs) with diameters of 10 nm, a tensile strength of 150 GPa have already been determined, which is very close to the theoretical limit^12^. Furthermore, for CNT fiber bundles, a tensile strength up to 80 GPa was reported^13^. The reason for these large differences in the theoretical and the measured tensile strength of fibers made from brittle materials like carbon and ceramics is, that only one defect leads to a catastrophic failure. A promising approach to reduce the probability for occurring defects in a defined fiber volume, is to reduce the diameter^14,15^. This phenomenon was already described for glass fibers by Griffith in 1921^16^. Flores et al*.* reported similar effects for SiCN ceramic fibers made from melt-spun oligosilazanes (OSZ). For example, a tensile strength of 800 MPa was measured for fibers with diameters of 90 µm, but for the same fibers with a diameter of only 30 µm a tensile strength of 1600 MPa was achieved^17^.

A promising technique to produce fibers with strongly reduced diameters in the nanometer scale is electrospinning. During electrospinning a suitable polymer is dissolved in a solvent and pumped through a syringe, where the solution is charged because of the friction with the cannula and accelerated by an electric field. On its way to the collector, the solvent evaporates and fibers with a diameter of several hundred nanometers are obtained and deposited on a collector. Usually, a flat collector is used, to produce nonwovens^18^. In principle, this technique is suitable to process almost all soluble polymers^18^ and was used in the past for example for carbon and ceramic precursors like PAN^19–22^, OSZ^23,24^ and siloxanes^25^.

Previously our groups developed highly flexible electrospun C/SiCON nonwovens from polymer blends of PAN and the OSZ^26^. In addition to extraordinary electrothermal properties, the nanofiber nonwovens were highly oxidation resistant. The reason for this combination of properties was the unique “sea-island” nanostructure where the ceramic interfaces protected the carbon from oxidation. However, this material has so far only been produced as nonwovens in which fibers were randomly laid onto each other. Because of their anisotropic structure fibers can withstand significantly higher loads in the direction of tension ^14^. Spinning into parallel, continuous multifibrillar fibers, could therefore achieve significantly higher tensile strengths and enable textile applications, for example as reinforcing fibers.

Aligned fibers can be collected on a fast-rotating collector. This technique was used by different research groups for producing PAN fibers. Stretching and twisting of such aligned fibers followed by pyrolysis provided yarns with a tensile strength of up to 1.7 GPa^27,28^. However, the length of the fiber bundles is limited by the diameter of the wheel, therefore only fibers with a length of several centimeters were obtained.

**Figure 1:**
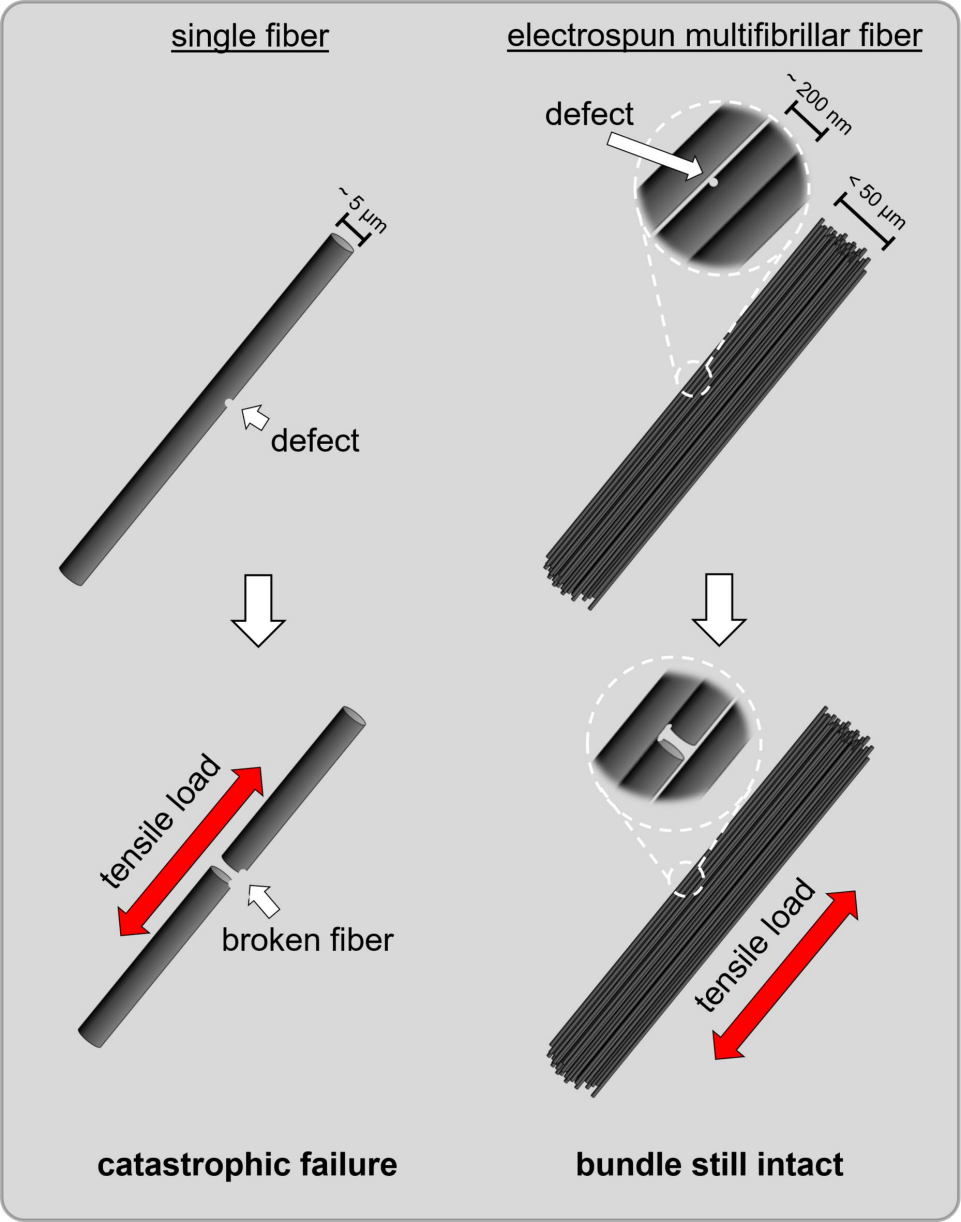
Schematic comparison of a defective single carbon or ceramic fiber with a multifibrillar fiber consisting of thousands of nanofibers. In contrast to the single fiber, the failure of one of the nanofibers under tensile load does not lead to the failure of the whole bundle.

However, a novel electrospinning approach allows the processing of continuous multifibrillar fibers. In this method, the polymer solution is spun by two differently charged syringes and the nanofibers are caught by a rotating funnel and wound to a continuous multifibrillar fiber. Since the individual fibers are not perfectly aligned directly after spinning, the fibers are subsequently stretched by two rollers turning at different speeds^29–32^. Extremely strong and tough multifibrillar polymer fibers were developed by additionally interconnecting the PAN nanofibers with polyethylene glycol (PEG) as a crosslinker via a click reaction^33^. The fibers were highly entangled and unaligned directly after electrospinning (as-spun), but by subsequent heating and stretching over the glass transition temperature the fibers were stretched to an almost perfect alignment of 99.6%^31^. In another publication electrospun multifibrillar PAN fibers were stretched with different ratios. Afterwards short fiber segments with lengths of serval centimeters were finally pyrolyzed to carbon fibers with tensile strengths of up to 1.1 GPa^29^. However, a stabilization step or optimization of the pyrolysis towards a continuous process was not carried out. In principle, this approach is extremely promising for producing ceramic and carbon fibers with extraordinary mechanical properties. As discussed before, because of the Griffith principle,^16^ such fibers with smaller diameters should have higher tensile strengths. Additionally, because of the multifibrillar nature of these fibers, it is assumed that the failure of some single filaments due to defects would not lead to a catastrophic failure of the multifibrillar fiber, as illustrated in Fig. 1. SiC fiber rovings, for example, usually consist of 3000 individual fibers, each with a diameter between 5 and 10 µm^9,34^. Using electrospinning, it would be possible to produce multifibrillar fibers from thousands of nanofibers, which would mean a significant reduction in linear density and is very interesting for lightweight construction. Particularly for fiber-reinforced plastics, it is important that the fibers are bound to the matrix as strongly as possible so that the strength of the fibers can be transferred to the matrix. Due to the extremely large surface to volume ratio of the multifibrillar fibers, a decisive improvement could also be expected for this application. Another important aspect for high temperature applications is the already proven increased oxidation stability of C/SiCON compared to pure carbon. However, so far only short fibers^35,36^ or nonwovens^26^ and no continuous multifibrillar fibers have been developed.

Therefore, the aim of this study was to create advanced, parallel aligned, continuous multifibrillar fibers with a high tensile strength composed of carbon and in particular of C/SiCON^24^ In order to adjust our approach as close as possible to a commercial fiber production and achieve good mechanical properties, all manufacturing steps including spinning, thermal stabilization, and pyrolysis, were carried out in an optimized continuous process.

## Experimental section

### Materials

The polyacrylonitrile copolymer (PAN) was obtained from Dolan GmbH (Germany) (copolymer with max. 8% methyl acrylate and methallylsulfonate according to the datasheet; number average molar mass (M_n_) = 95,000; registration No. 26658–88-8). The oligosilazane Durazane 1800 (OSZ) was acquired from Merck (Germany). Dicumylperoxide (DCP) was ordered from Sigma-Aldrich (Germany). *N,N´-*dimethylformamide (DMF) 99.99% and 99.9% acetone were shipped from Thermo Fisher Scientific GmbH (Germany). All materials were used as received, without further purification.

### Electrospinning and stretching of polymer fibers

Continuous yarns were obtained by yarn electrospinning following a procedure as published previously^33^. The solution for electrospinning was prepared by dissolving PAN powder and the OSZ Durazane 1800 in DMF solution with acetone according to Table S2. The continuous yarns were fabricated using a homemade setup comprising two syringe pumps, a high-voltage DC power supply, a poly(vinyl chloride) (PVC) funnel (8.0 cm in diameter) with a motor controller and a yarn winder collector with 2 cm in dimeter. The solution was loaded into two syringes capped with metal needles, respectively (controllable feed rate of 0.5 mL h^-1^ by two syringe pumps), which were connected separately to the positive and negative electrodes of the DC power supply. After adjusting the angle (13 degree of inclination), distance (40 cm) and altitude (perpendicular distance to the plane of the end of funnel: 2 cm) of these two syringes, high voltages (positive pole: +12 kV; negative pole: − 12 kV) were applied to the two needle tips respectively, resulting in positively and negatively charged continuous fibrils. At first, by the force of electric field, these two oppositely charged fibrils flew to the end of the funnel with 1500 rpm rotation speed and a fibril membrane would be formed. Followed by switching on the winder collector of 13 rpm rotation speed, the membrane was dragged by a pre-suspended yarn which was connected with the winder collector. Then a rotodynamic fibril cone could be formed above the funnel. Simultaneously, helical form fibrils in the fibril cone were pulled up in a spiral path. Due to the cone maintained by the continuous helical form fibrils, a polymer yarn with continuous and twisted form was prepared from the apex of the fibril cone and winded around the collector. The whole electrospun yarn process was operated with 10–15% humidity.

### Stabilization and pyrolysis to continuous C and C/SiCON fibers

For the continuous stabilization process eight fibers each were combined to a roving. The rovings were stabilized continuously under tension in air in a tubular furnace with three heating zones (Pyrolus AT). For the three stabilization runs, the furnace was programmed as follows:

I. 130–150–170 °C, II. 190–210–230 °C, III. 230–250–250 °C. Subsequently, the continuous pyrolysis was performed under tension at 1200 °C in N_2_ atmosphere (Nabertherm RHTC 80-710/15, Germany). The gas flow rate was set to 250 ml min^-1^. After pyrolysis the fibers were cut into smaller pieces for the analytical investigations.

### Thermogravimetric analysis and differential scanning calorimetry

Thermogravimetric measurements were performed using a STA 449 F5 Jupiter system (Netzsch, Germany). For this purpose, 5 to 8 mg samples were placed in an Al_2_O_3_ crucible. The heating rate was fixed to 5 K min^-1^ in air respectively nitrogen and the samples were heated to the respective maximum temperature. No hold time or special cooling rate was executed.

### Scanning electron microscopy

For the SEM images a Zeiss Sigma 300 VP (Gemini, Germany) scanning electron microscope equipped with a field emission cathode, a secondary electron (SE2) and an Inlens detector was used by an acceleration voltage of 3 kV and a working distance between 5 and 6 mm. Before the measurement small fiber samples were cut in liquid nitrogen. The pieces were fixed with a sample holder with a conductive double-sided carbon tape. The samples were subsequently sputter-coated with an 8 nm gold layer by a Cressington 108 auto sputter coater.

### ATR-FTIR spectroscopy

For the ATR-FTIR analysis a Tensor 27 system (Bruker, Germany) equipped with an ATR unit with a diamond crystal was used. After a background measurement, the samples were ground to a fine powder with a mortar and pressed against the measuring diamond to receive a suitable signal. The measurements took place in a wavenumber range of 4000–400 cm^-1^ at a resolution of 5 cm^-1^. 32 measurements were averaged per sample to obtain higher signal-to-noise ratios. After the measurement, the received data were saved as a .csv file and plotted graphically using Origin 2022 software.

### Raman spectroscopy

A combined Raman-imaging/scanning force microscope system (WITEC ALPHA 300 RA + , Germany) with WiTec Control FIVE 5.3 software was used for RAMAN measurements. The laser is equipped with a UHTS 300 spectrometer combined with a back-illuminated Andor Newton 970 EMCCD camera (Resolution: ca. 300–400 nm (lateral) and 900 nm (z) with 100 × objective). The measurements were carried out at an excitation wavelength of λ = 532 nm and a laser power of 1 mW with 50 accumulations with an integration time of 0.5 s pixel^−1^. The samples were fixed on a glass slide with a tape. A slight stress was applied to prevent movement or vibration of the fiber. After adjusting the focus on the fibers at 100 × magnification, the Raman spectrum was recorded. A cosmic ray removal and a baseline correction were applied for all spectra. The measured data were fitted with the GaussAmp function in Origin 2022.

### Transmission electron microscopy

TEM measurements were performed by a JEM-2200FS TEM (JEOL Corporation, Japan) at an acceleration voltage of 200 kV. For the sample preparation a bundle of 5 to 10 fibers were glued together with EPO-TEK 375 epoxy glue (Epotek, Germany) and afterwards put into EPO-TEK 301 epoxy resin (Epotek, Germany). The embedded fibers were cut with ultramicrotomy (Leica, Germany) to thicknesses of 50 nm at room temperature.

### Linear density

The linear density of the multifibrillar fibers (Table S3) were measured by weighing fiber bundles with a defined length and calculated by Eq. 1:

1 $$D=\frac{W}{L}$$

where the D is the linear density (tex = g km^-1^), W is the weight and L is the length of the multifibrillar fibers. The weight of the C and C/SiCON multifibrillar fibers with a length of 10–30 cm was measured by an ultramicro balance (Sartorius MSE2.7S-000-DM Cubis, capacity of 2.1 g, readability of 0.0001 mg, Germany).

### Determination of the density

The densities (Table S4) were determined using a helium pycnometer AccuPyc II 1340 (micromeritics, USA) with a 1 cm^3^ sample cup. To obtain a sufficient sample mass, electrospun nonwovens were used for the density measurements. The preparation of nonwovens is explained in detail elsewhere^26^. Between 0.02 and 0.04 g of each samples were used for the measurement. The mean value was calculated, from 10 measurements.

### Single fiber tensile strength

The tensile strengths were determined using a tensile tester (zwickiLine Z0.5, BT1-FR0.5TN.D14, Zwick/Roell, Germany) with a clamping length of 10 mm, a crosshead rate of 5 mm min^-1^ at 25 °C and a pre-tension of 0.001 N. The load cell was a Zwick/Roell KAF TC with a nominal load of 20 N. The fibers were glued to a paper frame according to german DIN1007 for single fibers tensile strength measurements (test length 25 mm). The multifibrillar fiber tensile tests were performed by a testing software belonging to the device for multifibrillar fiber shape cross-section calculation, while the linear density and density of the specimen material were input parameters. After the tensile test measurement, quantitative analysis of the tensile strength and Young’s modulus was carried out by Origin 2022 software.

### Electron beam treatment

An electron accelerator MB10-30MP (Mevex Corp., Stittsville, Canada) was used for electron beam treatment. The beam energy was 10 MeV and the total doses used were 300 – 1500 kGy. The dose was applied in partial steps of approximately 25 kGy to prevent heating of the samples over 60 °C. The nominal dose per path was determined by means of a calibrated graphite calorimeter. The polymer samples were sealed in a thin PVC-foil before irradiation. Samples were placed on an aluminum cooling block, kept at 35 °C operating temperature, to quickly dissipate the energy (heat) introduced by the irradiation. The fibers were wound onto a 10 mL syringe and a gentle nitrogen flow was adjusted through the inside and outside of the syringe to cool the sample during the treatment. The syringes were rotated by 90° after a quarter of total dose to account for differences in the dose depth profile and to ensure a homogeneous irradiation.

### Gel fraction

To determine the gel fraction of the crosslinked polymers, PAN or the solution of PAN/OSZ-40 in DMF listed in Table S1 were dried under reduced pressure at 70 °C for four days. After the electron beam treatment with different doses, 0.30 g of each polymer sample was dissolved in DMF for two days. Finally, the insoluble amount was then removed by filtration, washed, dried and weighted. The gel fraction was determined according to Eq. 2:

2 $$G=\frac{{m}_{2}}{{m}_{1}} \cdot 100$$

where *G* is the gel fraction in %, *m*_1_ is the mass of the polymer before dissolution, and *m*_2_ is the mass of the insoluble fraction after filtration.

### Melting tests

0.2 g each of the polymer powders of PAN and crosslinked OSZ were pressed to pellets (1 cm radius) with 400 bar using a hydraulic hand press (Enerpac, USA) with an HPS-2 /0,7A hydraulic system (Yale, Germany) and self-made steel molds. The synthesis of the crosslinked Durazane 1800 is explained in detail elsewhere^40^. The PAN/OSZ-40 pellets were prepared from the spinning solution in Table S2, poured into a glass mold, and dried in a vacuum drying oven at 70 °C for four days. Subsequently, the samples were treated with electron beam dose from 0 to 1000 kGy. Two samples were placed on top of each other, pressed together with a 200 g weight (~ 6200 Pa) and heated to 250 °C in air with a heating rate of 2 K min^-1^. The experiments were performed at least three times each.

## Results and Discussion

### Processing of fibers

The electrospinning process of the multifibrillar fibers was carried out using a special electrospinning approach and has been described in detail before^33^. For the preparation of the spinning solutions, PAN and the oligosilazane (OSZ) were dissolved in DMF and acetone. The solution was filled into two syringes, placed opposite to each other. Both solutions were continuously pumped from the syringes, one needle acted as a positive electrode, the other was negatively charged. A rotating funnel was located between the syringes, through which the spun nanofibers were collected and wound to a collector on the top. The aim was to produce multifibrillar fibers with the largest possible content of OSZ, since a larger proportion of OSZ leads to a better oxidation stability^26,35,36^ For the electrospinning of multifibrillar fibers OSZ composition of 40 wt.%^33^ was chosen, to ensure a stable spinning jet without fragmentation. For larger OSZ ratios, the jet kept splitting up and no continuous fiber was produced. Additionally, pure PAN fibers for the processing of multifibrillar carbon fibers were spun.

**Figure 2:**
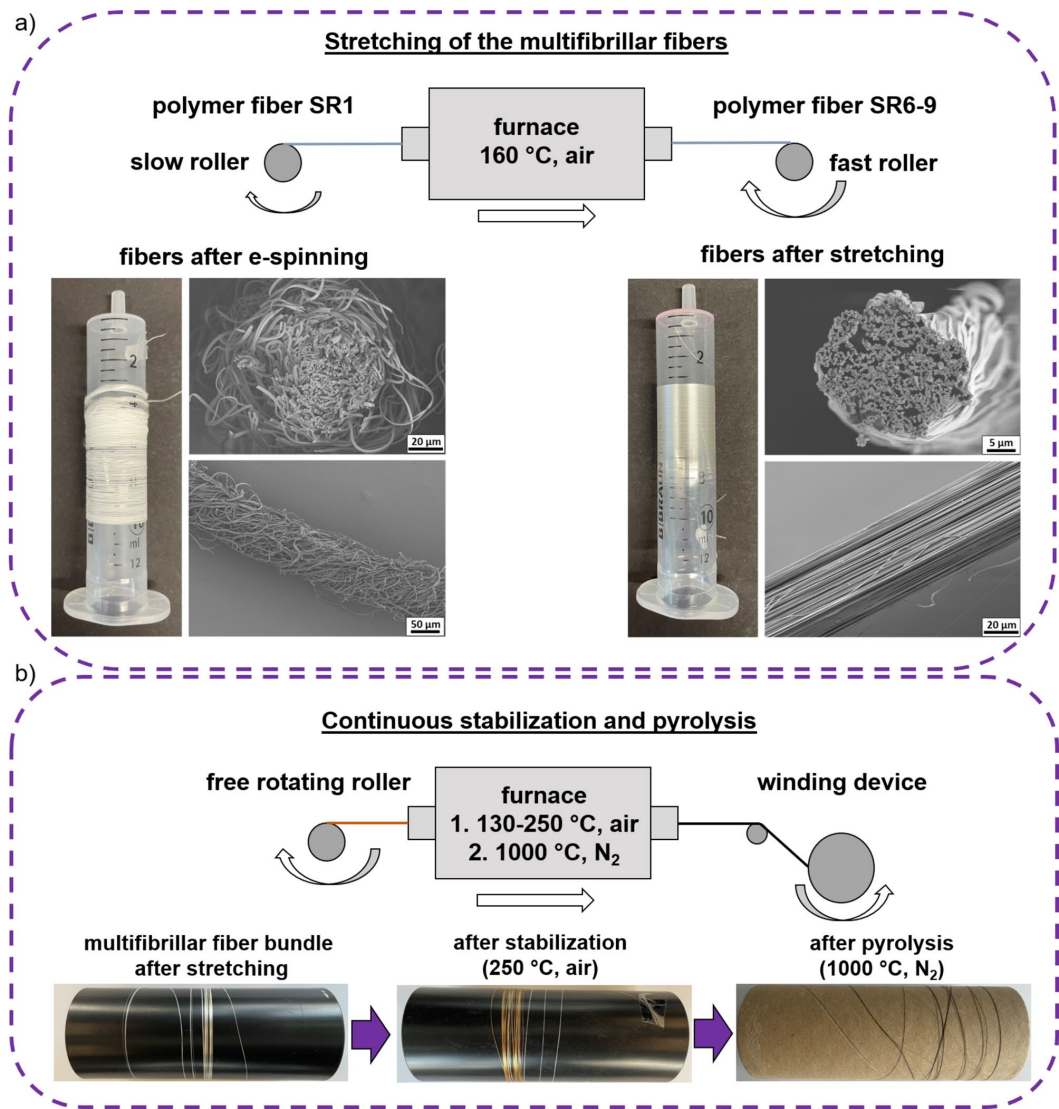
(**a**) Optical images and SEM micrographs of the stretching process of multifibrillar fibers from a disordered structure (SR1) to a highly aligned multifibrillar fiber (SR6-9). (**b**) Continuous oxidative stabilization at 250 °C in air and pyrolysis of the multifibrillar fibers at 1000 °C under N_2_-atmosphere.

Since the individual fibers were initially largely disordered, they were stretched and oriented. This was achieved by two rollers rotating at different speeds at 160 °C. The schematics, photographs and SEM micrographs of the fibers before and after stretching are displayed in Fig. 2 a. The stretching process is essential to obtain an almost parallel fiber alignment with a high orientation of the roughly counted 3000 individual nanofibers. Additionally, the stretching process led to reduced fiber diameters which should lead to better mechanical properties. Each multifibrillar fiber was stretched to the maximum stretch ratio (SR) without breaking. SR is defined as the length of stretched fibers divided by the length of the as-spun fibers (SR = 1 means unstretched). For pure PAN a maximum SR of 9 was achieved, whereas for fibers with 40 wt.% OSZ a SR of only 6 was reached (Fig. 2 a). A reason could be, that the OSZ was already partly crosslinked before or during the stretching process and therefore it was not possible to stretch as much. The resulting fibers are referred to as PAN or PAN/OSZ-40 in the polymer and C or C/SiCON-40 in the pyrolyzed state. The continuous stabilization and pyrolysis processes were optimized for PAN and PAN/OSZ-40 fibers. Therefore, 5 to 8 multifibrillar fiber bundles with lengths of 10 to 15 m were combined to a bundle. The stabilization (curing) step is crucial to obtain fibers that are mechanically and thermally stable enough to survive the subsequent pyrolysis without tearing. For the curing, the fibers were pulled through a tube furnace with three heating zones with a length of 30 cm each. The stabilization was performed in total in three runs from 130 to 250 °C. When the fibers were pulled through the furnace too quickly or at a maximum temperature lower than 250 °C, the fiber bundles always ruptured either during the stabilization or in the subsequent pyrolysis step.

The continuous pyrolysis was carried out at 1000 °C by drawing the fiber bundle through a tube furnace under nitrogen atmosphere with a self-made drawing machine and a free rotating roller as presented in Fig. 2 b.

### Investigation of the pyrolysis behaviour

**Figure 3:**
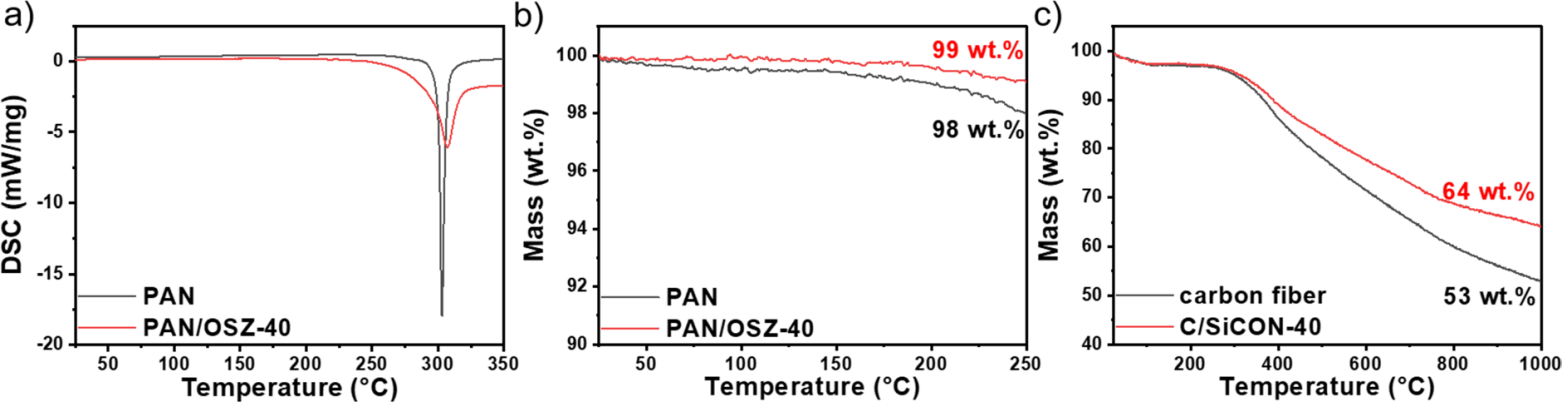
(**a**) DSC and (**b**) TGA measurements of PAN and PAN/OSZ-40 fibers during the stabilization process (heating rate 5 K min^-1^, air). (**c**) TGA measurements of the stabilized PAN and PAN/OSZ fibers during the pyrolysis process to carbon and C/SiCON-40 fibers (heating rate 5 K min^-1^, N_2_).

The curing and the pyrolysis behavior were investigated in detail by differential scanning calorimetry (DSC) and thermogravimetric analysis (TGA). During the stabilization process in air, the linear PAN chains react to a ladder-like structure by dehydrogenation and cyclization reactions^6,37,38^. These reactions are very exothermic leading to a strong signal in the DSC spectra for the two fiber types PAN and PAN/OSZ-40 (Fig. 3 a). Therefore, the heating rate during the stabilization process has to be very slow and should not exceed 250 °C, to prevent overheating of the fibers, which would lead to undesired reactions or damage the fibers^39^. The mass loss of 1 – 2 wt.% was negligible for PAN and for PAN/OSZ-40 during the stabilization process up to 250 °C (Fig. 3 b). During the stabilization, the PAN crosslinks to a ladder-like structure, which is accelerated by oxygen^1,3^. To further increase the degree of crosslinking and the yield of OSZ in the pyrolysis, dicumyl peroxide (DCP) was added to the OSZ as a radical initiator. DCP is known to start to crosslink OSZ at temperatures at 100 °C via vinyl polymerization and hydrosilylation during the stretching process, which avoids the release of volatile oligomers of the OSZ^40–43^. The actual mass loss of PAN and OSZ only starts at higher temperatures during the pyrolysis process (Fig. 3 c). The yield in the TGA measurement was slightly higher for C/SiCON-40 (64 wt.%) than for the pure carbon fiber (53 wt.%). The ladder-like structure of the crosslinked PAN still contains a large amount of nitrogen, which is released by the formation of HCN and N_2_ during the pyrolysis process between 200–1000 °C, leading to the formation of sp^2^ hybridized carbon^6,7^. The further crosslinking of the OSZ component occurs via dehydrocoupling and transamination reactions above 250 °C, with the release of H_2_ and NH_3_. In the range of 400–800 °C the precursor is transformed into an amorphous SiCON ceramic, by the formation of mainly H_2_ and CH_4_^42^. Thus, the PAN/OSZ-40 material, was converted to amorphous carbon and to an amorphous SiCON ceramic by the pyrolysis, resulting in a C/SiCON hybrid fiber^26^.

### Chemical characterization of the fibers

**Figure 4:**
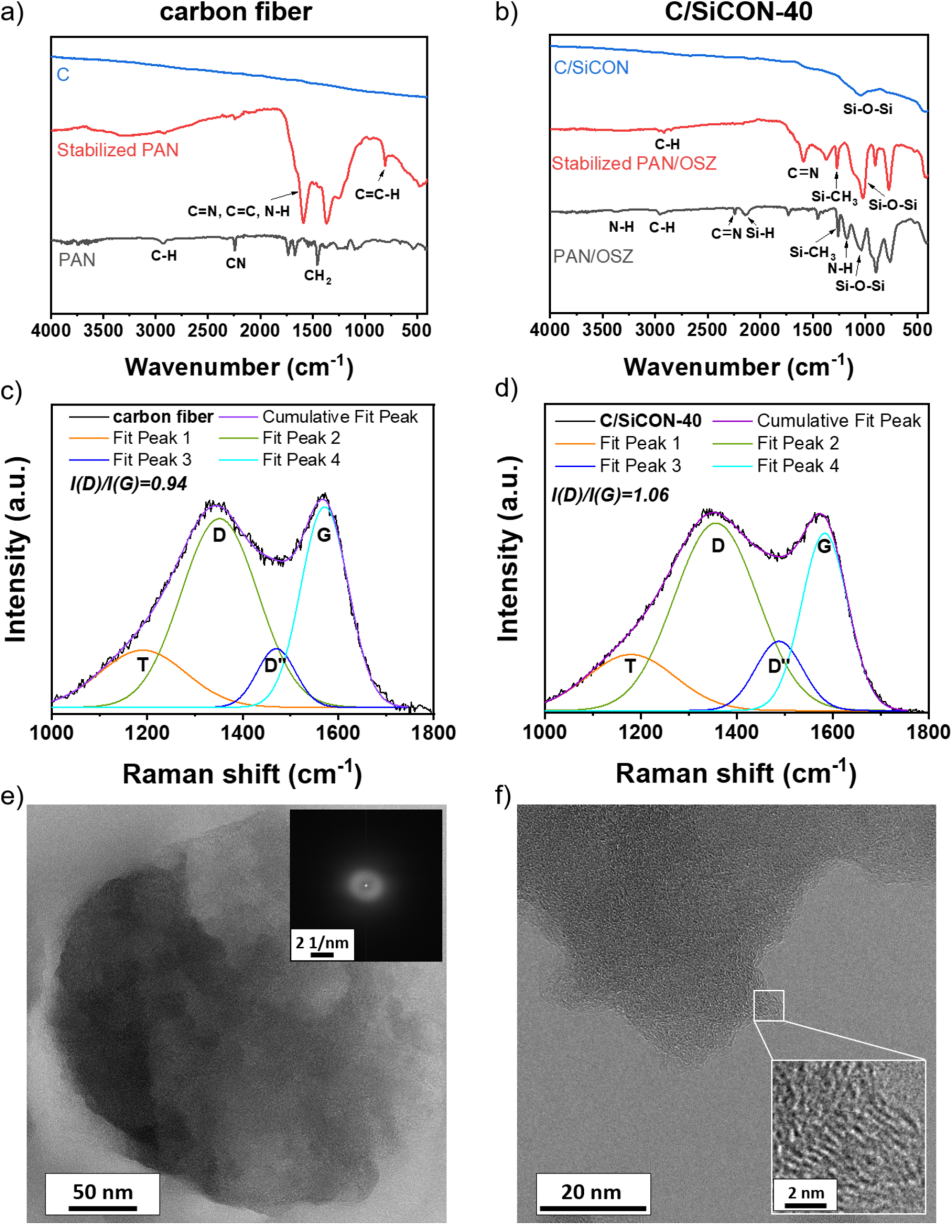
FTIR measurement from (**a**) carbon and (**b**) C/SiCON-40 multifibrillar fibers in the polymer, stabilized and pyrolyzed state. Raman analysis from (**c**) C and (**d**) C/SiCON-40 multifibrillar fibers in pyrolyzed state fitted with a Gaussian function and integrated area ratios^26^. TEM micrographs of C/SiCON-40 multifibrillar fibers in pyrolyzed state showing (**e**) amorphous phases with the corresponding FFT and (**f**) higher magnifications with graphitic carbon structures (inset shows the magnified graphitic-like structure).

Investigations of the PAN with attenuated total reflectance-fourier-transformed infrared spectroscopy (ATR-FTIR) confirmed that during the stabilization up to 250 °C the C≡N (2245 cm^-1^) groups were converted to C = N and C = C (1575 cm^-1^) bonds by cyclization reactions as can be seen in Fig. 4 a^1,8^. For the PAN/OSZ blend (Fig. 4 b), additionally N–H (3377 cm^-1^), Si–H (2139 cm^-1^), Si-CH_3_ (1263 cm^-1^) groups were detected belonging to the structure of the OSZ Durazane 1800 (Fig. S1). Since the silazane chains reacted with oxygen from air or were partially hydrolyzed from humidity in the air during the spinning process, additional Si–O-Si (1047 cm^-1^) groups were also detected^44,45^. The stabilization in air introduced more oxygen into the fiber which is clearly evident from the intensity increase in the Si–O-Si absorption band. Additionally, crosslinking reactions like hydrosilylation and dehydrocoupling proceeded in the OSZ, which is the reason for the less intensive Si–H and N–H absorption bands^42^. After pyrolysis, all organic groups disappeared and only Si–O-Si signals were detected for the C/SiCON-40 fibers. The Raman analysis delivered broad D and G bands with slightly higher *I*(D) to *I*(G) ratios of 1.06 for C/SiCON-40 fibers than for pure C with 0.94. This indicates a higher disordering of the carbon phase for C/SiCON-40 compared to pure C (Fig. 4 c and d). Transmission electron microscope (TEM) revealed that the microstructure of the C and C/SiCON-40 fibers consisted mainly of amorphous phases, confirmed also by the diffuse ring of the fast fourier-transformation (FFT) micrograph (Fig. 4 e). With higher magnifications some small graphitic carbon structures were also detected (Fig. 4 f). The mainly amorphous structure with free carbon phases was already described for similar materials after pyrolysis at temperatures of 1000 °C^26,35,36^.

The analytical results agree well with previous studies from our groups for similar materials from electrospun nonwovens, which are chemically identical to the multifibrillar fibers presented here^26^. The hybrid fibers in the nonwovens consisted of ceramic phases (SiO_4_, SiC_x_O_y_, SiN_x_O_y_, and SiC_x_N_y_) in a carbon matrix. The X-ray diffraction (XRD*)* and Raman analysis revealed the typical signals of amorphous carbon in the pyrolyzed fibers as well. ^13^C and ^29^Si solid state nuclear magnetic resonance (NMR) revealed the expected signals of sp^2^ carbon and a complex mixture of different SiO4, SiC_x_O_y_, SiN_x_O_y_, and SiC_x_N_y_ environments. The elemental analyses confirmed the presence of oxygen, which was introduced during the electrospinning and thermal stabilization in air.

### Investigation of the mechanical properties

**Figure 5:**
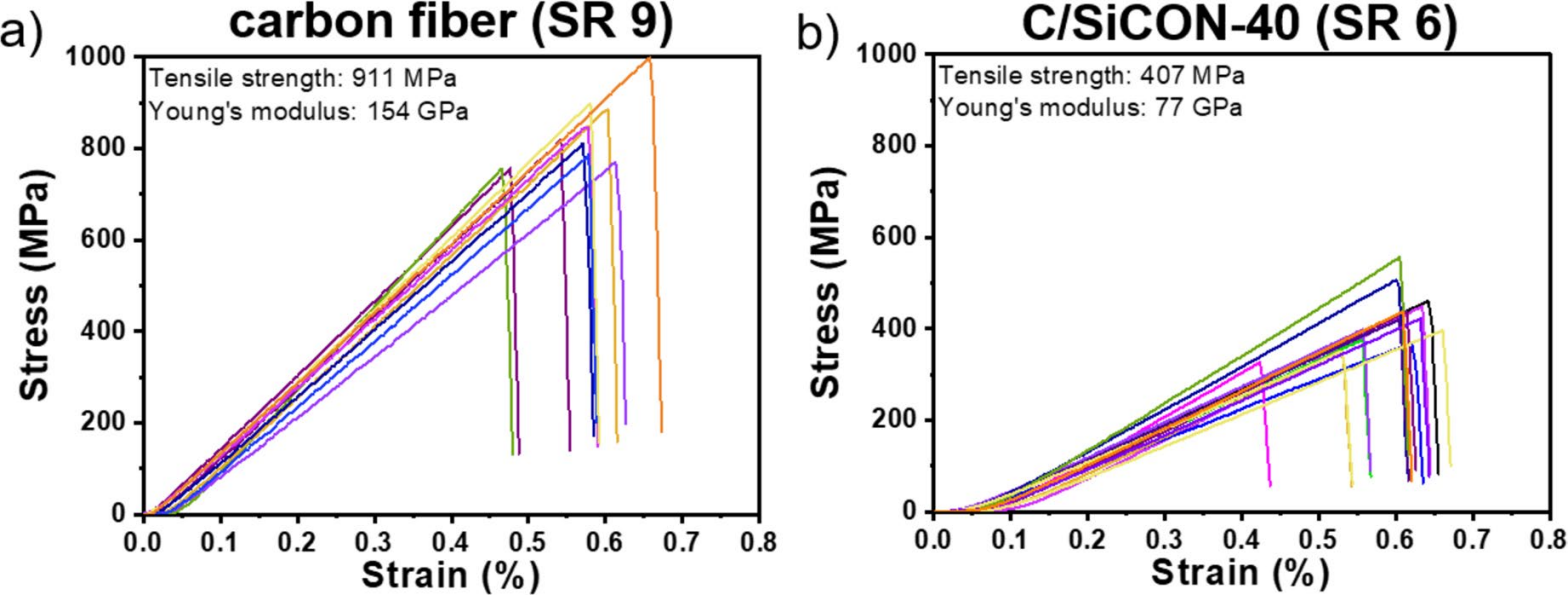
(**a**) Single fiber tensile tests of carbon multifibrillar fibers with a stretch ratio of 9. (**b**) Single fiber tensile tests of C/SiCON-40 multifibrillar fibers with a stretch ratio of 6.

To investigate the mechanical properties of the multifibrillar carbon and C/SiCON-40 fibers, single fiber tensile tests were performed (Fig. 5). The determined tensile strength of the multifibrillar carbon fibers with values of 911 ± 132 MPa and a Young’s modulus of 154 ± 50 GPa were much better than those of the C/SiCON-40 hybrid fibers with a tensile strength of 407 ± 73 MPa and a Young’s modulus of 77 ± 17 MPa. A first reason for the lower tensile strength of C/SiCON-40 was the smaller SR of 6, compared to SR 9 for carbon fibers. Therefore, the C/SiCON-40 fibers were less oriented and stretched, which led also to thicker multifibrillar C/SiCON-40 fibers with diameters of 40.7 ± 3.1 µm in comparison to the respective carbon fibers with diameters of 21.0 ± 3.5 µm. Similarly, the C/SiCON-40 individual nanofibrils had a larger diameter of 333 ± 41 nm compared to 269 ± 35 nm for the carbon fibers.

**Figure 6:**
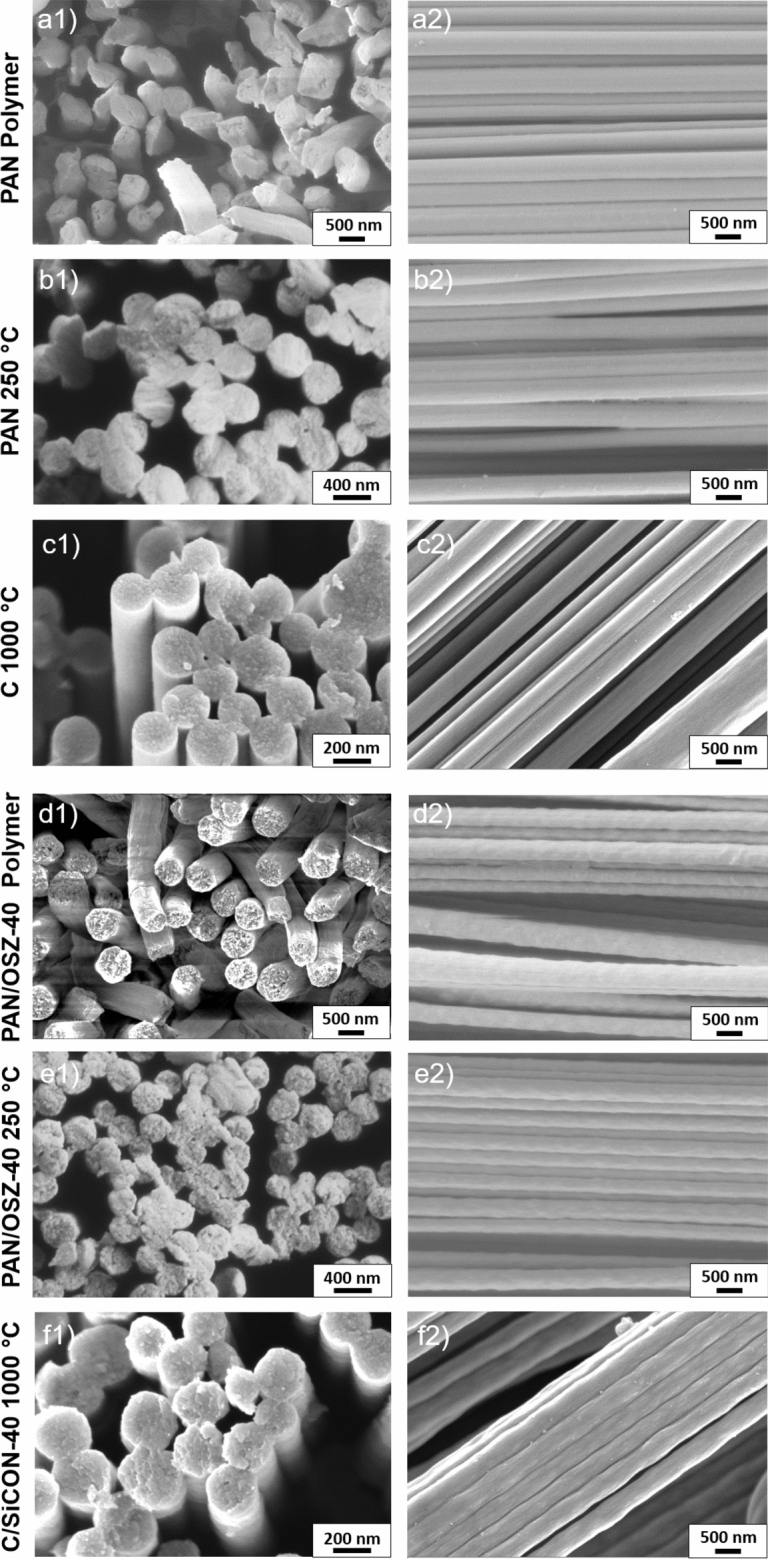
SEM micrographs of the PAN (**a**–**c**) and C/SiCON-40 (**d**-**f**) multifibrillar fibers in polymer condition, after stabilization at 250 °C and after pyrolysis at 1000 °C.

In addition, the fiber surface of the hybrid fibers was slightly rippled (Fig. 6 f) and not as smooth as for the carbon fibers (Fig. 6 c). As already described, the OSZ crosslinks during the stretching process at 160 °C and is therefore not as stretchable as the still not crosslinked PAN, which leads to a bulging morphology of each nanofiber. However, such a rough, uneven fiber structure of the individual nanofibers in the multifibrillar C/SiCON-40 fibers usually leads to a severe deterioration of the mechanical properties.

Another remarkable limitation of the tensile strengths of both fiber types is shown by analyzing the cross-section SEM micrographs of the multifibrillar fibers. The individual nanofibers in the polymer state (Fig. 6 a and d) partly fused together during the stabilization process (Fig. 6 b and e). Due to the very large surface of the nanofibers, the pressure on the fibers during stretching and their immediate contact, the softening of the PAN was sufficient for the fibers to stick together. Because of the described effect, many of the nanofibers did not exist individually, which might have resulted in a total failure of the multifibrillar structure.

### Electron beam treatment of the fibers

Different approaches were studied to prevent the softening of the PAN and the fusion of the individual fibers. In a first set of experiments, it was attempted to isolate the nanofibers with a separating agent. For example, the fibers were extracted with a soxhlet apparatus in acetone to remove residual DMF and afterwards impregnated with silicone oil (flash point > 315 °C) to act as a spacer between the individual nanofibers during thermal stabilization step (Fig. S2).

Furthermore, the use of catalysts should reduce the crosslinking temperature to such an extent that the PAN does not soften during the thermal stabilization and thus the nanofibers can be prevented from sticking together. Therefore, various salts from Sn^46–49^, Zn^50^, Co^51–53^, Cu^49,54^ and KMnO_4_^49,53,55^ were added as catalysts, as already reported in literature. A positive effect was observed in DSC for SnCl_2_ and ZnAc_2_ (Table S1 and Fig. S3). But neither the use of separating agents nor catalytic crosslinking prevented the fusion of the fibers (Figs. S2 and S4) or led to improved tensile strengths as shown in Fig. S3.

A more promising approach to improve the mechanical properties by avoiding sticking of the nanofibers was the crosslinking of the hybrid fibers by electron irradiation. This approach was reported for pure PAN fibers^56–59^ as well as for OSZ^17,60^ and is used commercially for polycarbosilanes during the production of ceramic SiC fibers of the second and third generation^34,61–64^. The electron radiation leads to the formation of reactive radicals and functional groups in the polymer structure, which subsequently crosslinks with each other and consequently increases the degree of crosslinking of the polymers remarkably at low temperatures.

**Figure 7:**
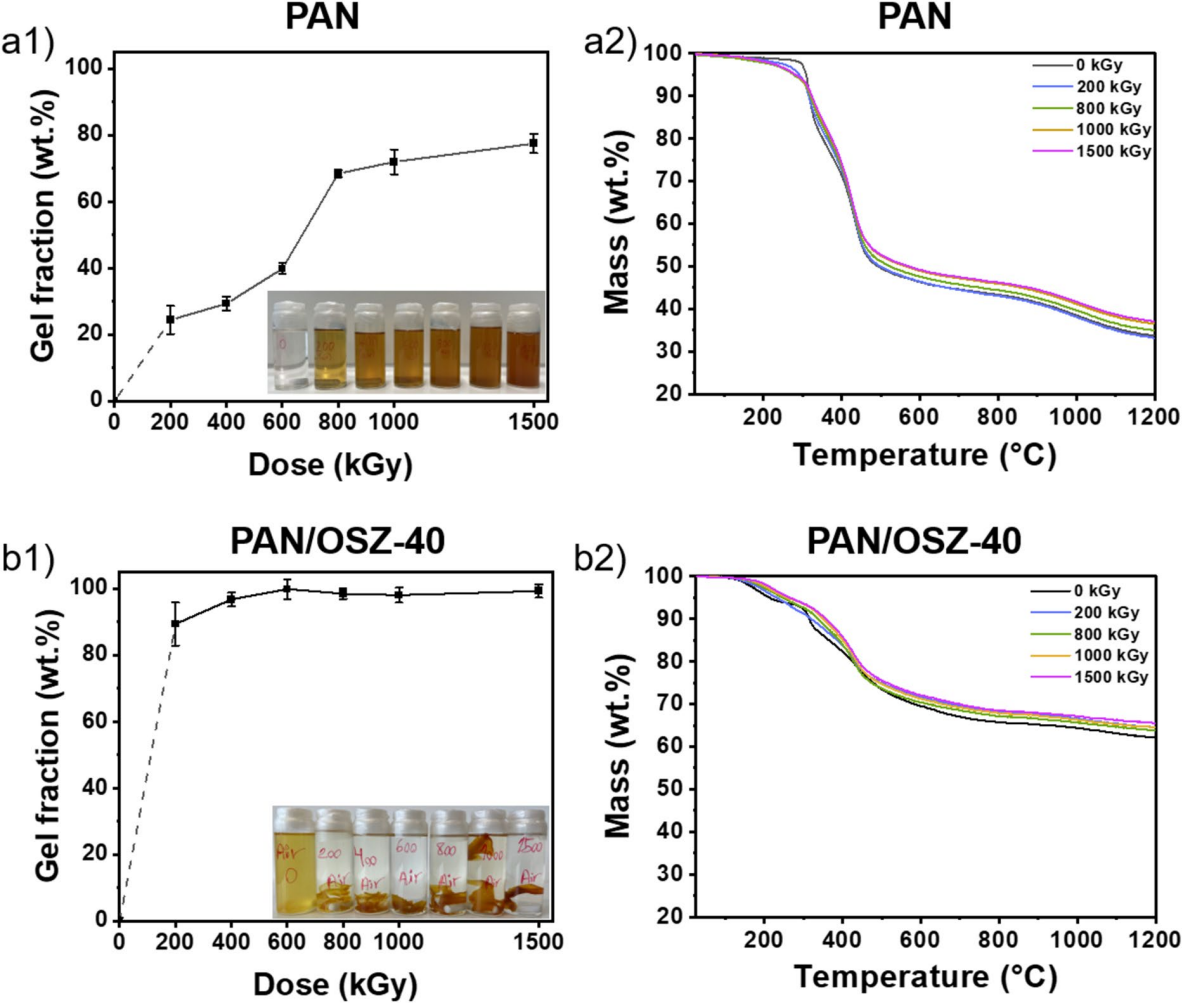
Gel fractions with optical images of the partly dissolved samples and TGA measurements (N_2_, 5 K min^-1^) of (**a**) PAN and (**b**) PAN/OSZ-40 with different doses of electron beam treatment.

To investigate and compare the crosslinking behavior, both pure PAN and blends of PAN/OSZ-40 were treated with different doses of electron radiation from 0 to 1500 kGy. For this purpose, the samples were dissolved in DMF after the treatment, filtered, and the percentage ratio of the mass of the dried, insoluble fraction relative to the untreated polymer was determined (Fig. 7 a1 and b1). As expected, with higher irradiation doses a higher degree of crosslinking for all polymers was achieved. The crosslinked fraction for pure PAN increased slightly from 24 to 40% at irradiation doses from 200 to 600 kGy. The largest gain in crosslinking was detected after the irradiation dose was raised to 800 kGy. After the treatment between 800 and 1500 kGy, the crosslinked fraction increased slower from 68 to 77%. For the PAN/OSZ-40 blends, a very high crosslinked fraction of 89% was determined already at a very low electron beam dose of 200 kGy. Above a dose of 400 kGy the polymer blend was almost completely crosslinked. The TGA measurements of the different PAN samples and PAN/OSZ blends (Fig. 7 a2 and b2) confirmed previous results that the ceramic yield, increased with the radiation dose^60^. The reason for the increased yield is the additional crosslinking during irradiation, which reduced the amount of volatile oligomers.

**Figure 8:**
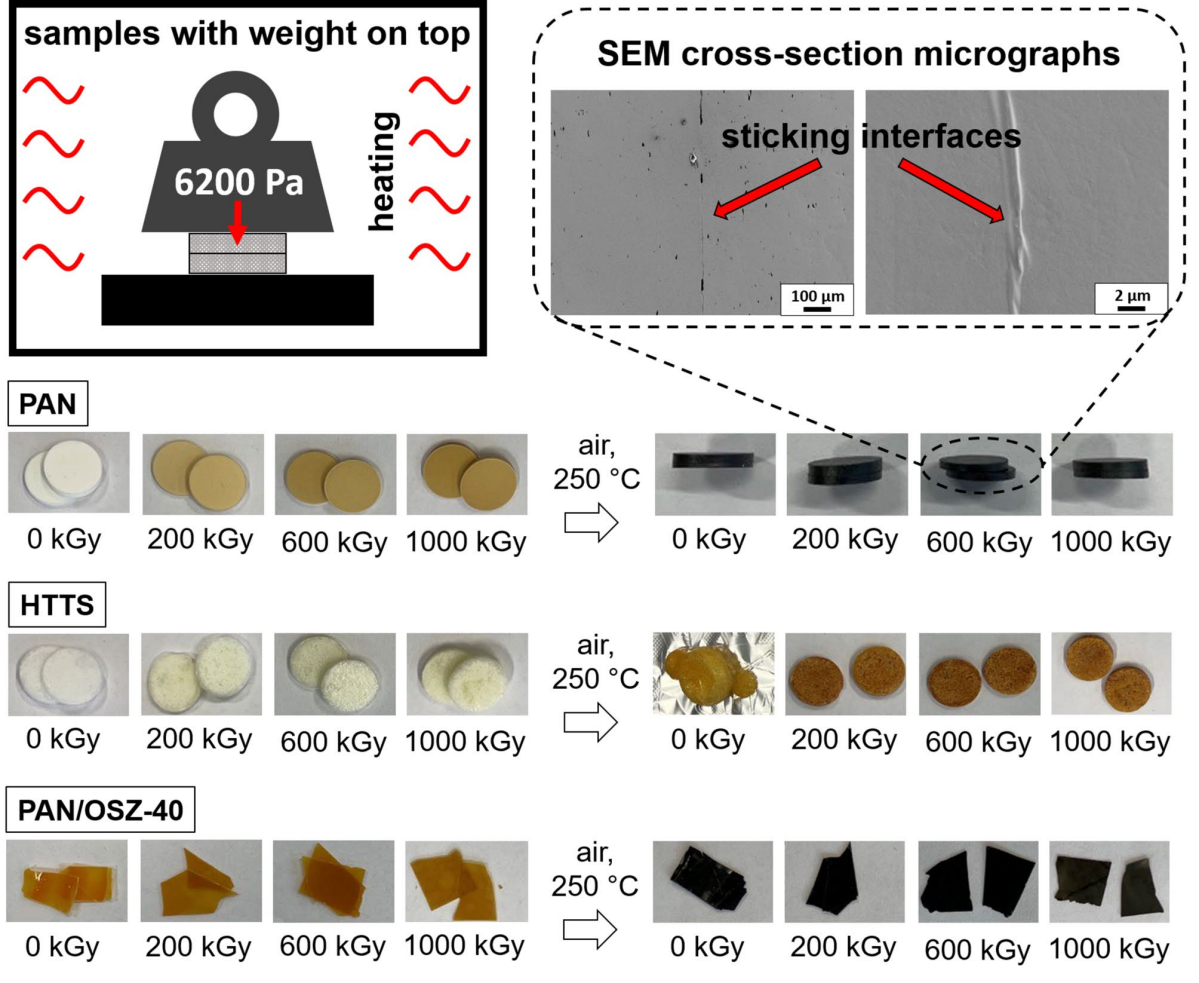
Schematic drawing of the sticking experiment of the irradiated polymer samples, and related optical images of the PAN, HTTS and PAN/OSZ-40 samples, treated with different doses of electron beam and SEM cross-section micrographs of the sticking PAN interfaces of the heated PAN samples.

In order to better understand the effects which led to the fusion of the nanofibers, pellets were pressed from powders of PAN and from catalytically polymerized OSZ (HTTS)^40^ or cast from PAN/OSZ-40 solutions, respectively. The resulting pellets were initially treated with different electron beam doses, placed on top of each other, and heated up to 250 °C in air according to the stabilization process of the fibers. As shown in Fig. 8, regardless of the electron beam treatment the pure PAN samples softened on the surface and stuck together. This is also clearly evident from the evaluation of the SEM micrographs of the cross-section. On the other hand, for polymerized OSZ (HTTS) melts, even electron beam treatment with low dose prevented sticking because of the high degree of crosslinking. As expected, the effect of electron irradiation on the crosslinking is therefore significantly higher for OSZ than for the pure PAN. The PAN/OSZ-40 pellets still fused together up to a radiation dose of 200 kGy, but this was no longer observed for higher doses.

**Figure 9:**
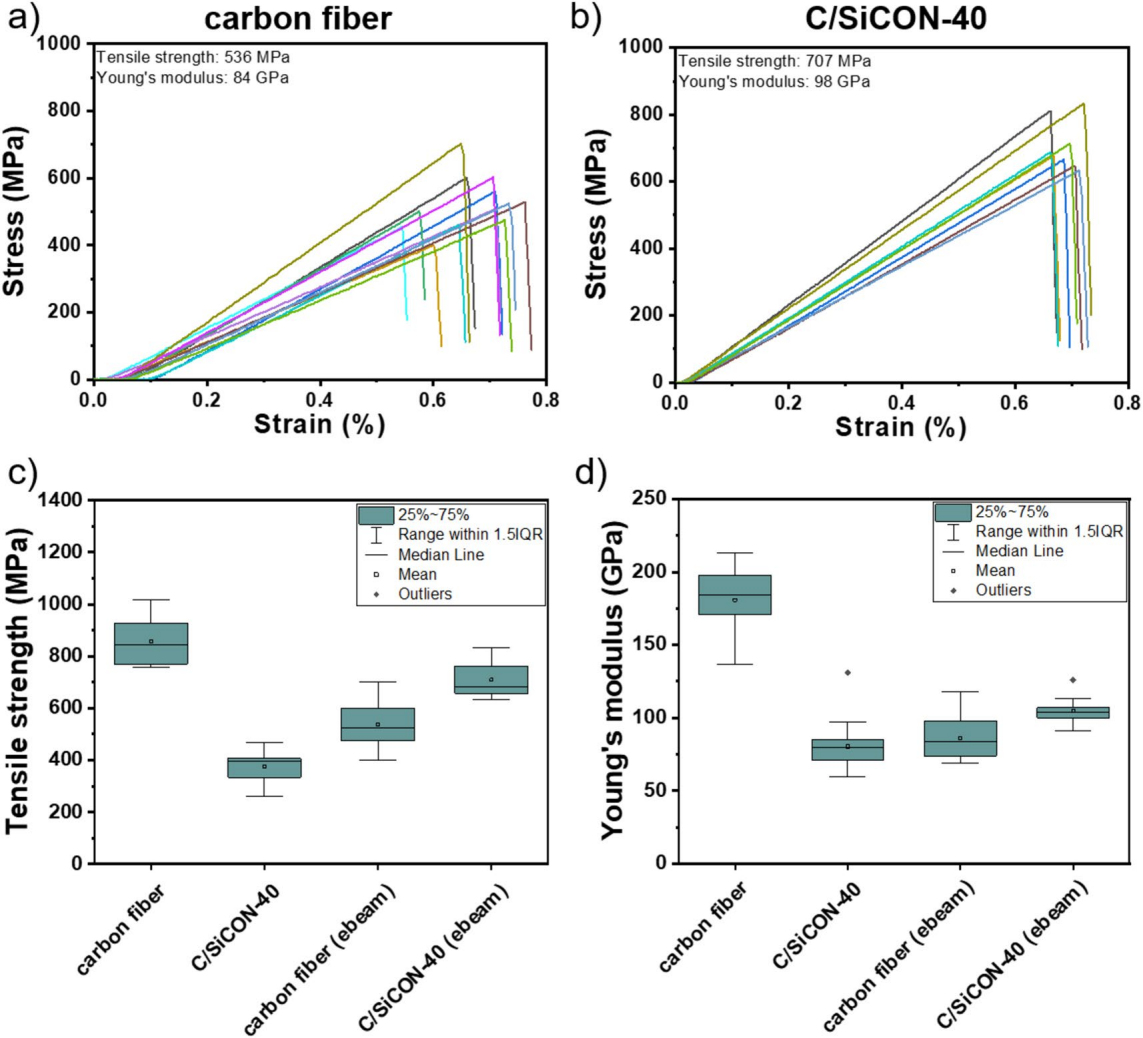
Single fiber tensile strength of (**a**) carbon multifibrillar fibers and (**b**) C/SiCON-40 multifibrillar fibers treated with an electron beam of a dose of 1000 kGy. Box-plots of the (**c**) tensile strengths and (**d**) Young’s modulus of C and C/SiCON-40 multifibrillar fibers with and without electron beam treatment.

Since the experiments to crosslink the polymers with electron irradiation were promising, the C and C/SiCON-40 multifibrillar fibers were treated with a high dose of 1000 kGy to ensure complete crosslinking. Afterwards the fibers were stabilized at 250 °C and pyrolyzed at 1000 °C as before. Despite the high electron beam dose, the SEM micrographs provided no evidence for damage of the PAN (Fig. S5) and PAN/OSZ-40 fibers (Fig. S6). But after further oxidative stabilization, the PAN fibers strongly fused together, whereas larger portions of the C/SiCON-40 fibers were found to be separated. Finally, the tensile strengths for the pyrolyzed electron beam treated multifibrillar carbon fibers (Fig. 9a) and the multifibrillar C/SiCON-40 fibers (Fig. 9 b) were determined and compared with the respective untreated fibers (Fig. 9 c and d). Because of the electron beam treatment, the tensile strength of the C/SiCON-40 fibers increased by 75% from 407 ± 73 MPa to 707 ± 80 MPa. Moreover, the Young’s modulus increased from 77 ± 11 GPa to 103 ± 10 GPa as well. In contrast, the electron beam curing of the carbon fibers, led to a strong decrease of 41% of the tensile strengths from 911 ± 190 MPa to 536 ± 85 MPa. Thus, it can be concluded that the additional crosslinking was not sufficient to reduce the sticking of the multifibrillar carbon fibers. Additionally, treatment by electron irradiation results in either degradation or crosslinking of a polymer structure^65–67^, and therefore it cannot be ruled out that defects are introduced into the fiber by the irradiation that led to a deterioration of the tensile strength.

Although the tensile strengths should be higher for the multifibrillar nanofibers due to the Griffith principle^16^ discussed before, the values are decreased by various factors. For example, even in the multifibrillar C/SiCON-40 fibers, sticking was only reduced, but not completely prevented. In addition, during the spinning process the fibers were collected and entangled by a rotating funnel as a collector^33^. Due to the subsequent stretching, the fibers were aligned very well, but entanglements within the multifibrillar fibers were still very likely. These led to lateral shear forces, in the brittle ceramic fibers, and thus severely limited the tensile strengths. In addition, for the determined tensile strengths it must be considered that the multifibrillar fibers are comparable with fiber rovings and not with single fibers. Fiber rovings generally have a significantly lower tensile strength than single fibers^68,69^. A first reason is friction between the individual fibers in the bundle, which is enhanced if weaker fibers break under low load, and additional friction occurs between the intact fibers and the fiber fragments^68–70^. Moreover, the breakage of individual fibers in the bundle caused a sudden load on the neighboring intact fibers, which results in their breakage even if these fibers would have withstood a corresponding uniform load^71^. Because the individual fibers have diameters in the nanoscale, it was not possible to prepare single-fiber tensile tests, which are normally used for mechanical fiber tests.

Furthermore, the low weight expressed by the linear density of the multifibrillar fibers has to be considered for the mechanical properties. The corresponding tex value (g km^-1^) of the multifibrillar carbon fibers was 0.33 tex and 1.04 tex for the multifibrillar C/SiCON-40 fibers. Commercial carbon fibers are in the order of 198 tex for rovings made of 3000 single fibers (TORAY T300 fibers)^9^ or ~ 200 tex for SiC ceramic rovings made of 1800 single fibers (Hi-Nicalon)^72^. The low inherent weight of the fibers in combination with the high values for tensile strengths shows the potential of the electrospun multifibrillar fibers. The unique combination of mechanical properties, the ability to produce these fibers continuously in an industrial process, and the previously published high oxidation stability of this material^26^, makes this approach highly promising for an advanced carbon and ceramic fiber class. It offers potential applications in a wide range of uses.

## Conclusions

In this work we presented for the first time continuous electrospun carbon and C/SiCON multifibrillar nanofibers, consisting of thousands of single filaments in the nanometer scale. The initially disordered polymer fibers were stretched almost perfectly to parallel aligned multifibrillar nanofibers. Subsequently, the fibers were continuously stabilized at 250 °C in air and pyrolyzed at 1000 °C in nitrogen atmosphere to carbon and ceramic C/SiCON nano-fibers, which consist of an amorphous carbon structure containing nano-scaled ceramic phases. Because of fiber sticking the tensile strength was only 407 MPa and the Young’s modulus 77 GPa for the C/SiCON-40 multifibrillar fibers, whereas for the respective multifibrillar carbon fibers a higher tensile strength of 911 MPa and a Young’s modulus of 154 GPa were determined. However, an additional electron beam treatment for the multifibrillar C/SiCON fibers led to a significant increase in both in the tensile strength up to 707 MPa and in the Young’s modulus up to 103 GPa. In contrast, for the pure carbon the tensile strength decreased to 536 MPa and the Young’s modulus to 98 GPa fibers probably due to the generation of defects within the fiber structure during irradiation.

Considering the low linear density of the multifibrillar C/SiCON nanofibers (~ 1 tex) compared to conventional C and SiC fiber bundles (~ 200 tex), the high mechanical properties of the fibers become evident. Since all steps were carried out in a continuous approach, this method can be extended and applied to any fiber length and is suitable for an industrial scale and setup. Because of the high tensile strength in combination with the low inherent weight, the extremely large surface to volume ratio and the already shown flame stability (C/SiCON) the multifibrillar fibers are of high interest as reinforcing component for example in the field of advanced fiber-reinforced plastics. In addition, the high-temperature and oxidation stability (C/SiCON) demonstrated in previous studies also enables use in composite materials for high temperature applications with carbon and ceramics as a matrix. The future research activities are focused on further improving the processing of the multifibrillar fibers to obtain significantly better mechanical properties.

### Supplementary Information

Supplementary Information.

## Data Availability

The datasets used and/or analyzed during the current study available from the corresponding author on reasonable request.
