# Supplementary material for: Novel multifibrillar carbon and oxidation-stable carbon/ceramic hybrid fibers consisting of thousands of individual nanofibers with high tensile strength
Source: Sci Rep. 2024 Aug 5;14:18143. doi: 10.1038/s41598-024-68794-w (PMC11300710; doi:10.1038/s41598-024-68794-w)
Supplement: Supplementary file 1 — Supplementary Information. [file 41598_2024_68794_MOESM1_ESM.docx]

**Novel multifibrillar carbon and oxidation-stable carbon/ceramic hybrid fibers consisting of thousands of individual nanofibers with high tensile strength**

Jakob Denk,^a,^ Xiaojian Liao,^b,*^ Wolfgang Knolle,^d^ Axel Kahnt,^d^ Andreas Greiner,^b^ Stefan Schafföner,^a^ Seema Agarwal,^b,c,*^ and Günter Motz^a,^[[1]](#footnote-1)^*^

a Chair of Ceramic Materials Engineering, University of Bayreuth, Bayreuth 95440, Germany.

b Macromolecular Chemistry 2 and Bavarian Polymer Institute, University of Bayreuth, Bayreuth 95440, Germany

c Bavarian Center for Battery Technology (BayBatt), University of Bayreuth, Bayreuth 95440, Germany

d Leibniz Institute of Surface Engineering (IOM), Permoserstr. 15, 04318 Leipzig, Germany

**Fig. S1** Chemical structure of Polyacrylonitrile PAN and the oligosilazane Durazane 1800.


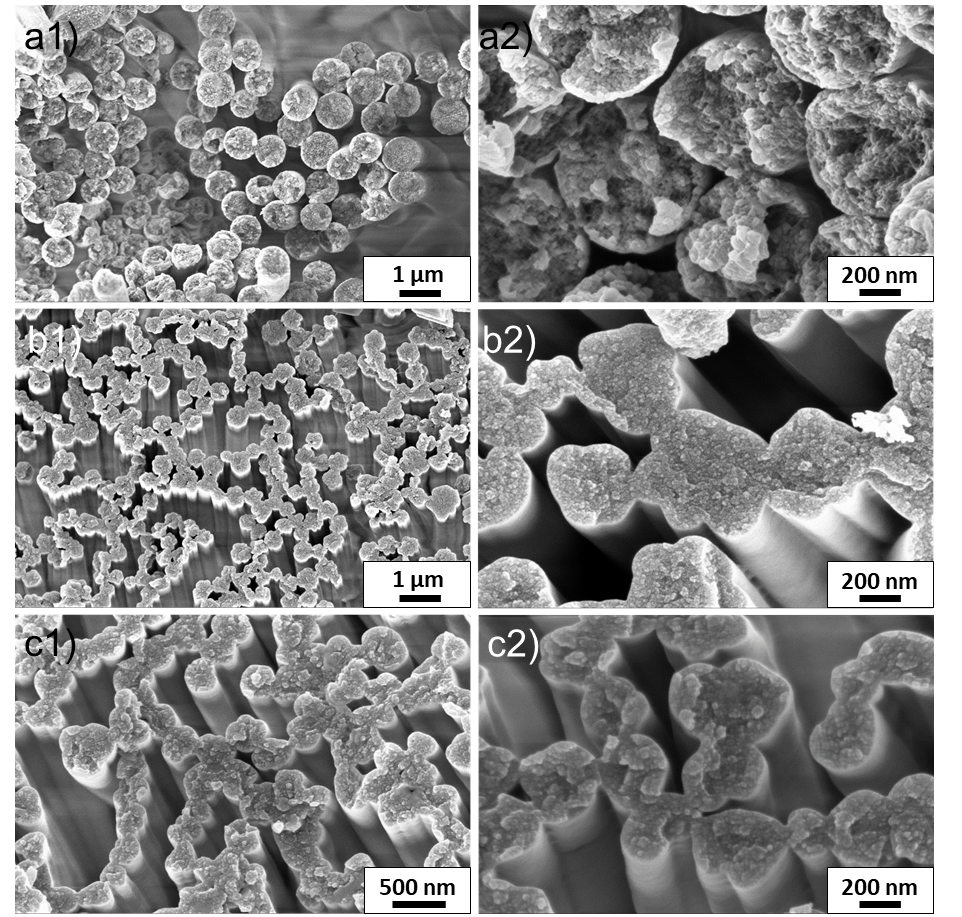
**Fig. S2** SEM images of PAN multifibrillar fibers after a) soxhlet extraction in acetone, b) soxhlet extraction and oxidative stabilization at 250 °C and c) immersing in silicon oil and oxidative stabilization at 250 °C

**Table S1** Peak location, onset and released energy of the crosslinking reaction of PAN with different catalysts.

| Catalyst | Peak  [°C] | Energy  [J g^-1^] | Peak Onset  [°C] |
| --- | --- | --- | --- |
| - | 310 | -1390 | 266 |
| SnCl_2_ | 285 | -635 | 167 |
| ZnAc_2_ | 292 | -756 | 205 |
| SnF_2_ | 312 | -720 | 266 |
| Co(acac)_3_ | 302 | -896 | 255 |
| CoCl_2_ | 293 | -716 | 246 |
| CuAc_2_ | 298 | -660 | 232 |


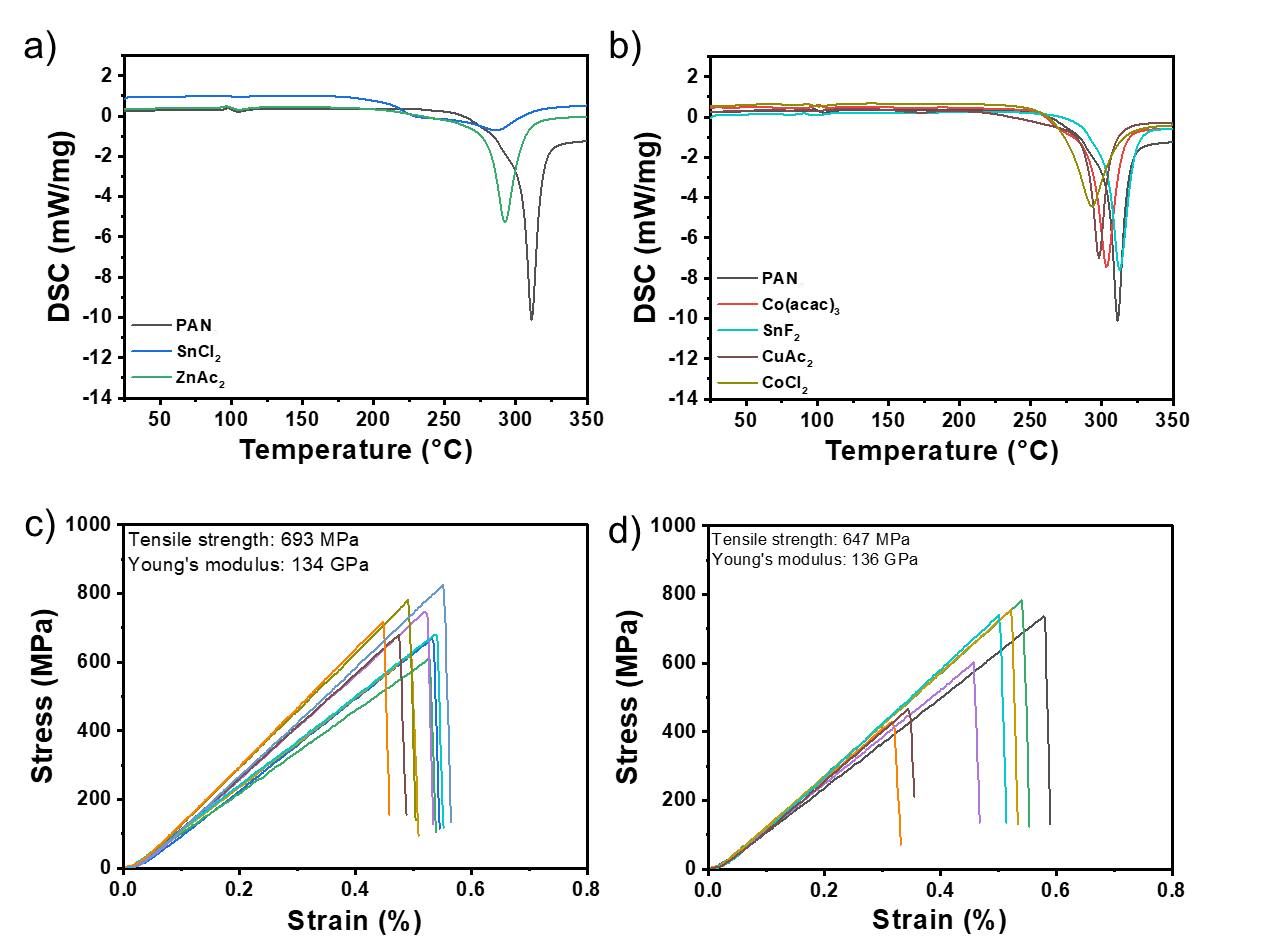


**Fig. S3** DSC measurements of the stabilization process of PAN with the catalysts a) SnCl_2_, ZnAc_2_ and b) Co(acac)_3_, SnF_2_, CuAc_2_ and CoCl_2_. Single fiber tensile strength of multifibrillar carbon fibers with catalysts c) SnCl_2_ and d) ZnAc_2_


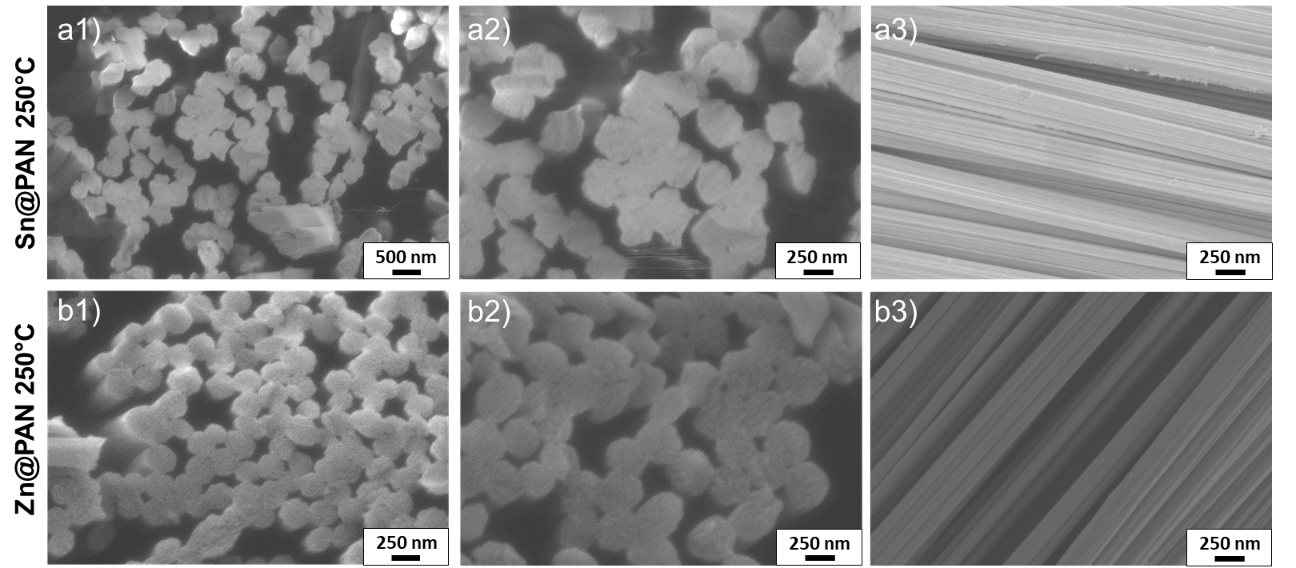


**Fig. S4** SEM images of PAN multifibrillar fibers with 3 wt.% of the catalysts a) SnCl_2_ and b) ZnAc_2_ and oxidative stabilization at 250°C.


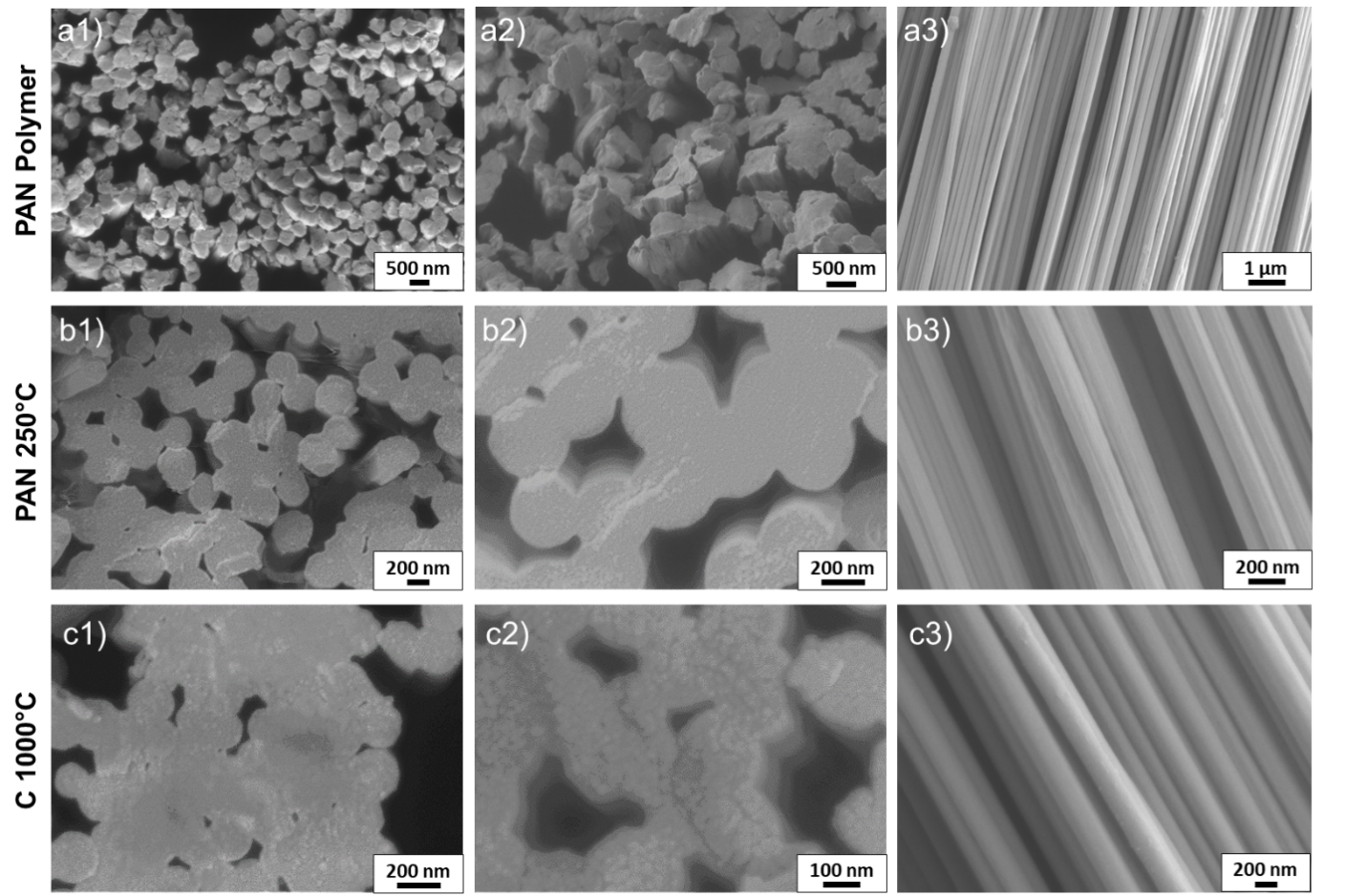


**Fig. S5** SEM images of carbon multifibrillar fibers after an electronbeam treatment of 1000  kGy in a) polymer condition, b) after stabilization at 250 °C and c) pyrolysis at 1000°C.


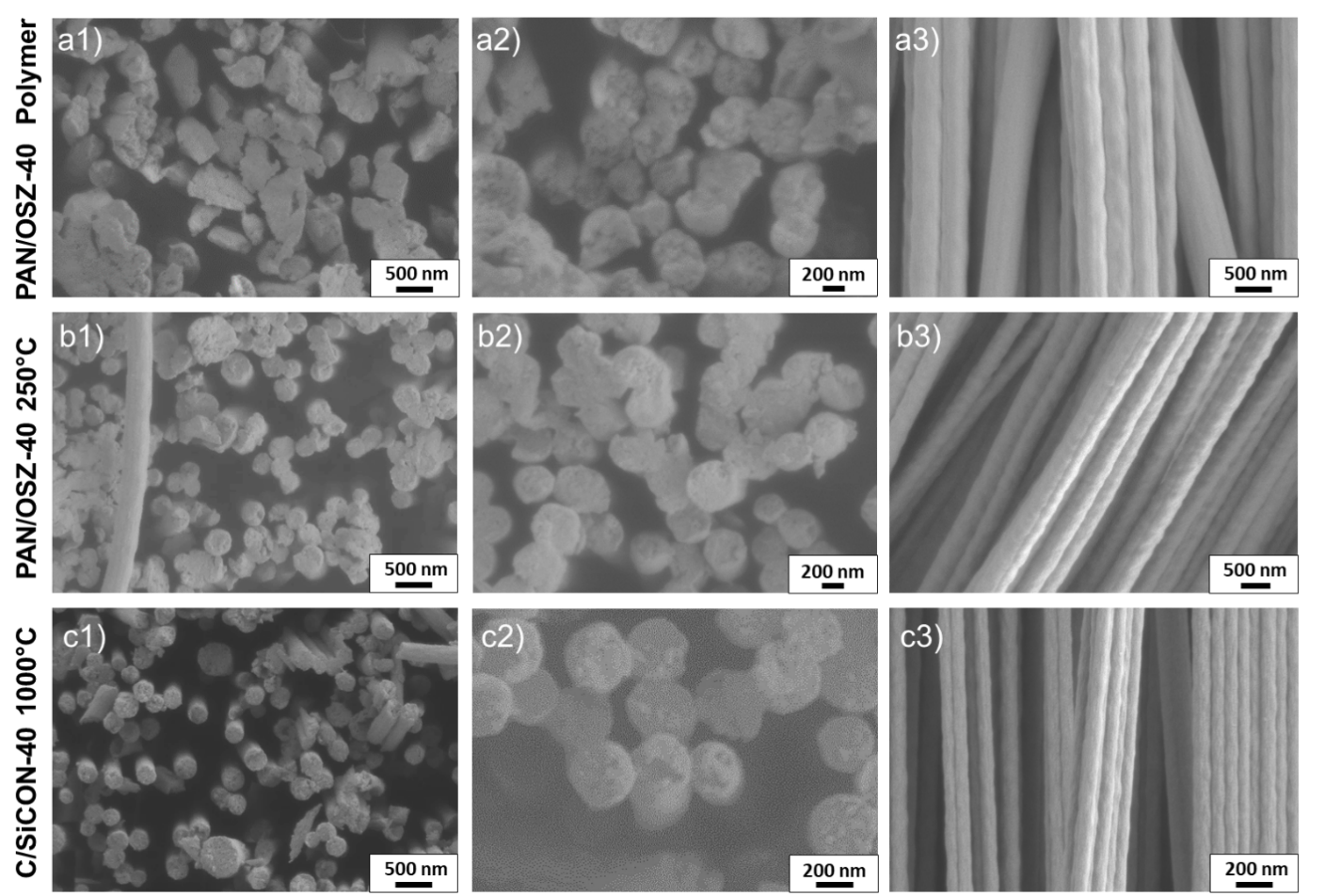


**Fig. S6** SEM images of C/SiCON-40 multifibrillar fibers after an electronbeam treatment of 1000 kGy in a) polymer condition, b) after stabilization at 250 °C and c) pyrolysis at 1000°C.

**Table S2:** Compositions of electrospinning solutions of PAN and PAN/OSZ-40 fibers. 3 wt.% DCP was added with respect to OSZ.

| Content  [wt.%] | PAN  [g] | OSZ  [g] | DMF  [g] | Acetone  [g] | SR |
| --- | --- | --- | --- | --- | --- |
| 0 | 1 | - | 4.70 | 0.96 | 9 |
| 40 | 1 | 0.67 | 6.03 | 0.67 | 6 |

**Table S3:** Linear densities of the various electrospun mulitfibrillar fibers.

| sample | Linear density  [tex] |
| --- | --- |
| carbon fiber SR9 | 0.33 |
| C/SiCON-40 SR6 | 1.04 |

**Table S4:** Measured densities of the various electrospun fibers.

| sample | Density  [g cm^-3^] |
| --- | --- |
| carbon fiber | 1.80 (± 0.045) |
| C/SiCON-40 | 2.00 (± 0.075) |

1. * Corresponding author. E-mail address: Guenter.Motz@uni-bayreuth.de (Günter Motz); Seema.Agarwal@uni-bayreuth.de (Seema Agarwal); xj_liao24@tju.edu.cn (Xiaojian Liao) [↑](#footnote-ref-1)
